# 1,5-Bis(piperidin-1-yl)-9,10-anthraquin­one

**DOI:** 10.1107/S1600536812050313

**Published:** 2012-12-19

**Authors:** Paweł Niedziałkowski, Elżbieta Wnuk, Anna Wcisło, Damian Trzybiński

**Affiliations:** a Faculty of Chemistry, University of Gdańsk, J. Sobieskiego 18, 80-952 Gdańsk, Poland

## Abstract

In the centrosymmetric title compound, C_24_H_26_N_2_O_2_, the piperidine ring adopts a chair conformation and is inclined at a dihedral angle of 37.5 (1)°to the anthracene ring system. In the crystal, adjacent mol­ecules are linked through C—H⋯π and π–π [centroid–centroid distances = 3.806 (1) Å] inter­actions, forming a layer parallel to the *bc* plane.

## Related literature

For general background to quinone compounds, see: Alves *et al.* (2004 ▶); El-Najjar *et al.* (2011 ▶); Czupryniak *et al.* (2012 ▶); Krohn (2008 ▶); Wannalerse *et al.* (2008 ▶). For related structures, see: Niedziałkowski *et al.* (2010 ▶, 2011 ▶); Wnuk *et al.* (2012 ▶); Yatsenko *et al.* (2000 ▶).
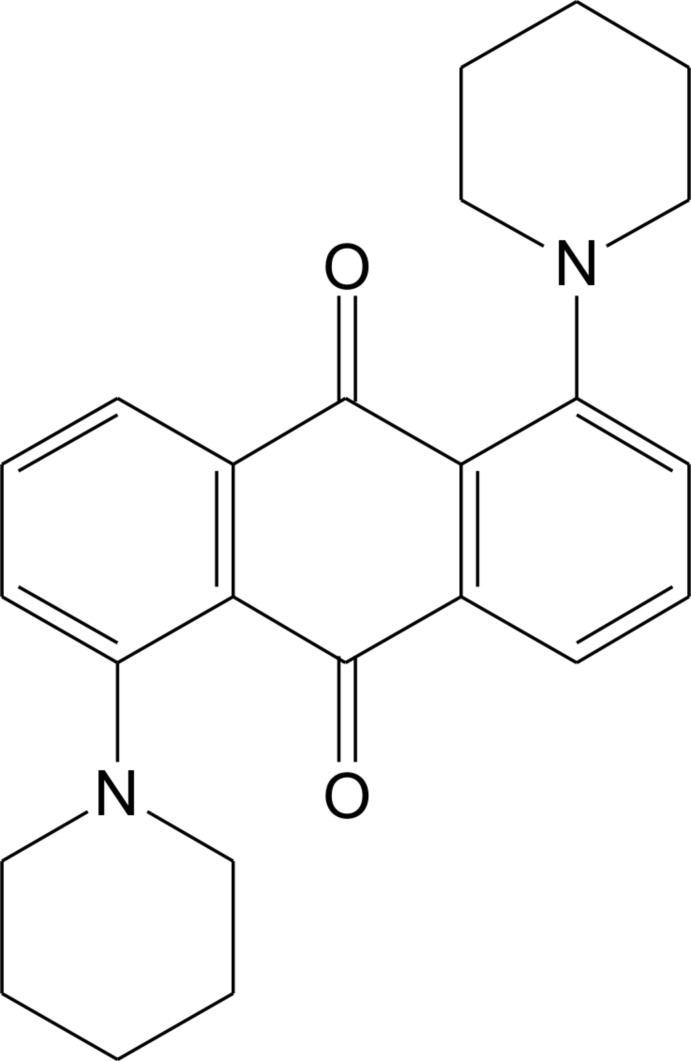


## Experimental

### 

#### Crystal data

C_24_H_26_N_2_O_2_
*M* *_r_* = 374.47
Monoclinic, 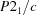

*a* = 10.9115 (4) Å
*b* = 7.0127 (2) Å
*c* = 12.5984 (5) Å
β = 97.819 (4)°
*V* = 955.05 (6) Å^3^
*Z* = 2
Mo *K*α radiation
μ = 0.08 mm^−1^
*T* = 295 K
0.45 × 0.22 × 0.05 mm

#### Data collection

Oxford Diffraction Gemini R Ultra Ruby CCD diffractometer
Absorption correction: multi-scan (*CrysAlis RED*; Oxford Diffraction, 2008 ▶) *T*_min_ = 0.909, *T*_max_ = 1.000
12625 measured reflections
1699 independent reflections
1274 reflections with *I* > 2σ(*I*)
*R*_int_ = 0.042

#### Refinement

*R*[*F* ^2^ > 2σ(*F* ^2^)] = 0.041
*wR*(*F* ^2^) = 0.102
*S* = 1.04
1699 reflections
127 parameters
H-atom parameters constrained
Δρ_max_ = 0.13 e Å^−3^
Δρ_min_ = −0.14 e Å^−3^

### 

Data collection: *CrysAlis CCD* (Oxford Diffraction, 2008 ▶); cell refinement: *CrysAlis RED* (Oxford Diffraction, 2008 ▶); data reduction: *CrysAlis RED*; program(s) used to solve structure: *SHELXS97* (Sheldrick, 2008 ▶); program(s) used to refine structure: *SHELXL97* (Sheldrick, 2008 ▶); molecular graphics: *ORTEP-3* (Farrugia, 2012 ▶); software used to prepare material for publication: *SHELXL97* and *PLATON* (Spek, 2009 ▶).

## Supplementary Material

Crystal structure: contains datablock(s) global, I. DOI: 10.1107/S1600536812050313/xu5662sup1.cif

Structure factors: contains datablock(s) I. DOI: 10.1107/S1600536812050313/xu5662Isup2.hkl

Supplementary material file. DOI: 10.1107/S1600536812050313/xu5662Isup3.cml

Additional supplementary materials:  crystallographic information; 3D view; checkCIF report

## Figures and Tables

**Table 1:** Hydrogen-bond geometry (Å, °) *Cg*2 is the centroid of the C1–C6 ring.

*D*—H⋯*A*	*D*—H	H⋯*A*	*D*⋯*A*	*D*—H⋯*A*
C2—H2⋯*Cg*2^i^	0.93	2.98	3.850 (2)	156

Symmetry code: (i) 
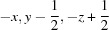
.

**Fig. 1.**
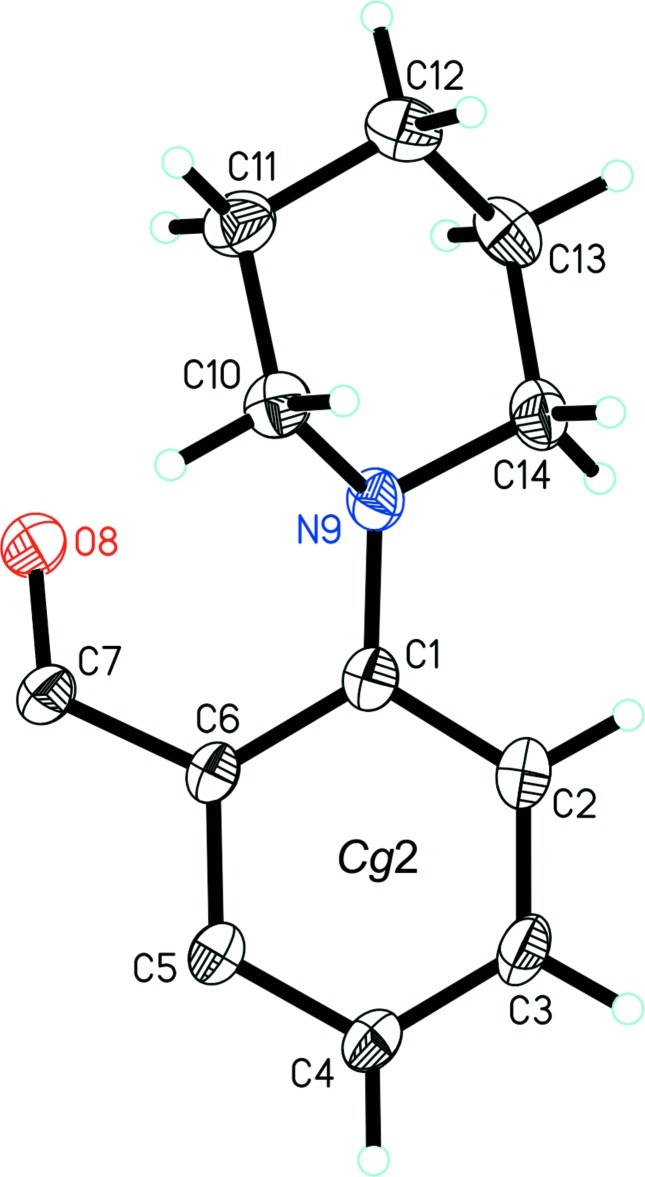
The asymmetric part of the unit cell of the title compound, showing the atom-labeling scheme. Displacement ellipsoids are drawn at the 25% probability level, and H atoms are shown as small spheres of arbitrary radius. Cg2 is the centroid of the C1–C6 ring.

**Fig. 2.**
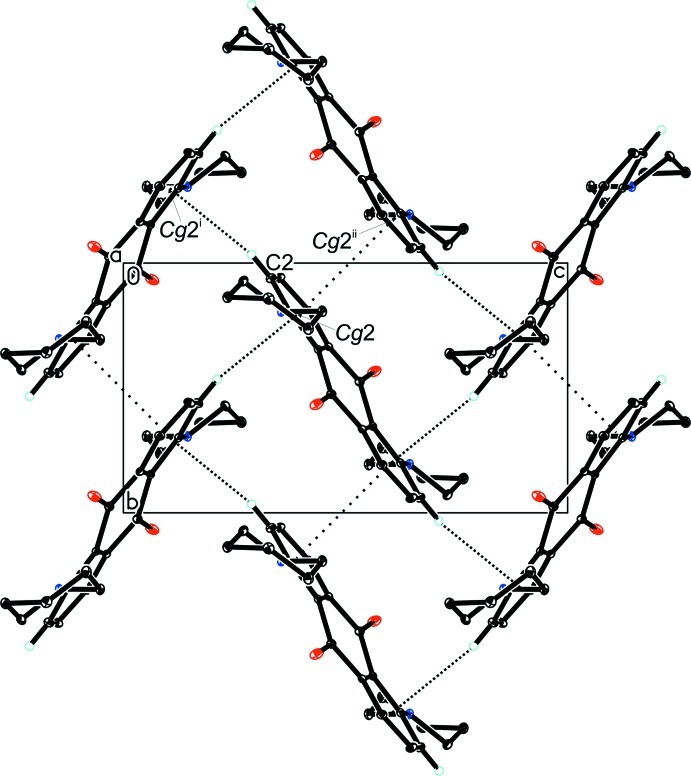
The arrangement of the molecules in the crystal structure. The C—H···π and π–π interactions are represented by dotted lines. H atoms not involved in interactions have been omitted. [Symmetry codes: (i) –x, y – 1/2, –z + 1/2; (ii) –x, –y, –z + 1.]

## supplementary crystallographic information

### Comment

Quinone and quinone-derived compounds are widely distributed in the environment. They occur in many plants as physiologically active substances participating in photosynthetic electron transport processes (El-Najjar *et al.*, 2011). Anthraquinones, both natural and synthetic, are coloring compounds with many applications in industry, mainly as pigments, food colorants and textile dyes. Some of the anthraquinone derivatives have been used for medical purposes as anticancer drugs and antitumor or antiviral agents (Alves *et al.*, 2004; Krohn, 2008). Finally, derivatives belonging to this group of compounds are applied in molecular and supramolecular chemistry as optical and electrochemical sensors (Czupryniak *et al.*, 2012; Wannalerse *et al.*, 2008). This wide variety of practical applications make anthraquinone derivatives an important object of research and natural target in organic synthesis.

The crystal structures of some 9,10-anthraquinone derivatives were described in our previous papers (Niedziałkowski *et al.*, 2010; Niedziałkowski *et al.*, 2011; Wnuk *et al.*, 2012). The purpose of this work is to report the crystal structure of 1,5-di(piperidin-1-yl)-9,10-anthraquinone.

The title compound has only half of molecule in the asymmetric part of the unit cell (Fig. 1). In the crystal structure, each half of molecule is arranged around an inversion centre located in the middle of the quinone ring. In the molecule of the title compound, likewise in other 9,10-anthraquinone derivatives (Niedziałkowski *et al.*, 2010; Niedziałkowski *et al.*, 2011; Wnuk *et al.*, 2012, Yatsenko *et al.*, 2000), deviation of planarity of the anthraquinone skeleton is observed. In case of the title compound, such distortion is found to be 0.0834 (3) Å. The piperidine rings adopt a chair conformation, with ring-puckering parameters *Q* = 0.5680 (18) Å, *Θ* = 178.23 (18)° and *φ* = 207 (6)°. The mean planes of piperidine and anthracene ring systems are inclined at a dihedral angle of 37.5 (1)°. The neighboring anthracene moieties are parallel or inclined at an angle of 63.3 (1)° in the crystal lattice. In the crystal, the adjacent molecules are linked by C—H···π (Table 2, Fig. 2) and π–π [centroid-centroid distances = 3.806 (1) Å] (Table 3, Fig. 2) interactions, forming a layer parallel to the *bc* plane.

### Experimental

To a solution of 1 g 1,5-bis(tosyloxy)-9,10-anthraquinone (1.823 mmol) in 50 ml of toluene 0.392 g of piperidine was added (4.775 mmol). The mixture was heated to 80°C. After 24 h the reaction mixture was cooled to room temperature. The resulting mixture was filtered and the solvent was removed under reduced pressure. The residue was dissolved in dichloromethane (200 ml) and washed with water (3 x 200 ml). The organic layer was dried over anhydrous MgSO_4_ and concentrated under reduced pressure to obtain a red solid residue. This solid was purified by flash column chromatography (SiO_2_, eluent: dichloromethane) to afford the 0.657 g (yield: 96%) of the title compound. Dark-red crystals suitable for X-ray investigations were grown from dichloromethane/methanol solution (1:1, v/v) (m.p. 207–208°C).

*Spectral data:*

^1^H NMR (CDCl_3_, 400MHz): δ (ppm): 1.628–1.686 (p, 2H, –N–CH_2_–CH_2_–CH_2_–CH_2_–CH_2_–, *J_1_*=5.6 Hz, *J_1_*=6.0 Hz, *J_2_*=5.8 Hz, *J_2_*=6 Hz, *J_3_*=11.8 Hz); 1.809–1.866 (p, 4H, –N–CH_2_–CH_2_–CH_2_–CH_2_–CH_2_–, *J_1_*=5.2 Hz, *J_1_*=5.6 Hz, *J_1_*=6.0 Hz, *J_2_*=5.4 Hz, *J_2_*=5.6 Hz, *J_2_*=5.8 Hz, *J_3_*=11.1 Hz); 3.137 3.164 (p, 4H, –N–CH_2_–CH_2_–CH_2_–CH_2_–CH_2_–, *J_1_*=5.2 Hz, *J_1_*=5.6 Hz, *J_2_*=5.4 Hz); 7.258–7.280 (d, 2H, H-2 Ar, H-4 Ar, *J_1_*=8.8 Hz); 7.528–7.568 (t, 2H, H-3 Ar, H-7 Ar, *J_1_*=*J_2_*=8,0 Hz); 7.807–7.826 (d, 2H, H-6 Ar, H-8 Ar, Hz, *J_1_*=7.6 Hz);

IR (KBr): 3434, 2940, 2855, 2813, 1648, 1582, 1423, 1381, 1226, 895, 710 (cm^–1^);

MALDI-TOF MS: m/z 376.3 [M+H]^+^ (MW = 374.20).

### Refinement

H atoms were positioned geometrically, with C—H = 0.93 Å and 0.97 Å for the aromatic and methylene H atoms, respectively, and constrained to ride on their parent atoms with *U*_iso_(H) = *xU*_eq_(C), where *x* = 1.2 for the aromatic and *x* = 1.5 for the methylene H atoms.

### Crystal data

**Table:**

C_24_H_26_N_2_O_2_	*F*(000) = 400
*M**_r_* = 374.47	*D*_x_ = 1.302 Mg m^−^^3^
Monoclinic, *P*2_1_/*c*	Mo *K*α radiation, λ = 0.71073 Å
Hall symbol: -P 2ybc	Cell parameters from 5096 reflections
*a* = 10.9115 (4) Å	θ = 3.3–29.2°
*b* = 7.0127 (2) Å	µ = 0.08 mm^−^^1^
*c* = 12.5984 (5) Å	*T* = 295 K
β = 97.819 (4)°	Plate, dark-red
*V* = 955.05 (6) Å^3^	0.45 × 0.22 × 0.05 mm
*Z* = 2	

### Data collection

**Table:**

Oxford Diffraction Gemini R Ultra Ruby CCD diffractometer	1699 independent reflections
Radiation source: Enhanced (Mo) X-ray Source	1274 reflections with *I* > 2σ(*I*)
Graphite monochromator	*R*_int_ = 0.042
Detector resolution: 10.4002 pixels mm^-1^	θ_max_ = 25.1°, θ_min_ = 3.3°
ω scans	*h* = −13→13
Absorption correction: multi-scan (*CrysAlis RED*; Oxford Diffraction, 2008)	*k* = −8→8
*T*_min_ = 0.909, *T*_max_ = 1.000	*l* = −15→15
12625 measured reflections	

### Refinement

**Table:**

Refinement on *F*^2^	Primary atom site location: structure-invariant direct methods
Least-squares matrix: full	Secondary atom site location: difference Fourier map
*R*[*F*^2^ > 2σ(*F*^2^)] = 0.041	Hydrogen site location: inferred from neighbouring sites
*wR*(*F*^2^) = 0.102	H-atom parameters constrained
*S* = 1.04	*w* = 1/[σ^2^(*F*_o_^2^) + (0.0491*P*)^2^ + 0.0697*P*] where *P* = (*F*_o_^2^ + 2*F*_c_^2^)/3
1699 reflections	(Δ/σ)_max_ < 0.001
127 parameters	Δρ_max_ = 0.13 e Å^−^^3^
0 restraints	Δρ_min_ = −0.14 e Å^−^^3^

### Special details

**Table:**

Geometry. All esds (except the esd in the dihedral angle between two l.s. planes) are estimated using the full covariance matrix. The cell esds are taken into account individually in the estimation of esds in distances, angles and torsion angles; correlations between esds in cell parameters are only used when they are defined by crystal symmetry. An approximate (isotropic) treatment of cell esds is used for estimating esds involving l.s. planes.

### Fractional atomic coordinates and isotropic or equivalent isotropic displacement parameters (Å^2^)

**Table:**

	*x*	*y*	*z*	*U*_iso_*/*U*_eq_	
C1	0.09986 (14)	0.19940 (19)	0.37606 (11)	0.0393 (4)	
C2	0.01598 (16)	0.0605 (2)	0.33222 (14)	0.0513 (4)	
H2	0.0428	−0.0338	0.2889	0.062*	
C3	−0.10447 (16)	0.0595 (2)	0.35124 (15)	0.0559 (5)	
H3	−0.1586	−0.0320	0.3184	0.067*	
C4	−0.14652 (15)	0.1917 (2)	0.41814 (13)	0.0472 (4)	
H4	−0.2273	0.1852	0.4338	0.057*	
C5	−0.06790 (13)	0.33481 (18)	0.46218 (12)	0.0383 (4)	
C6	0.05433 (13)	0.34603 (18)	0.43854 (11)	0.0368 (4)	
C7	0.12218 (14)	0.5249 (2)	0.46817 (13)	0.0430 (4)	
O8	0.21900 (12)	0.56569 (16)	0.43513 (12)	0.0720 (4)	
N9	0.22226 (11)	0.19390 (17)	0.35623 (10)	0.0436 (3)	
C10	0.32009 (14)	0.1930 (2)	0.44808 (12)	0.0499 (4)	
H10A	0.3308	0.0645	0.4763	0.060*	
H10B	0.2961	0.2742	0.5041	0.060*	
C11	0.44077 (15)	0.2628 (3)	0.41651 (14)	0.0588 (5)	
H11A	0.5047	0.2570	0.4780	0.071*	
H11B	0.4320	0.3946	0.3934	0.071*	
C12	0.47827 (16)	0.1425 (3)	0.32700 (15)	0.0609 (5)	
H12A	0.4974	0.0141	0.3527	0.073*	
H12B	0.5518	0.1958	0.3030	0.073*	
C13	0.37392 (16)	0.1369 (3)	0.23460 (14)	0.0578 (5)	
H13A	0.3628	0.2630	0.2032	0.069*	
H13B	0.3958	0.0507	0.1800	0.069*	
C14	0.25403 (16)	0.0721 (2)	0.27005 (14)	0.0537 (5)	
H14A	0.1884	0.0772	0.2099	0.064*	
H14B	0.2621	−0.0588	0.2948	0.064*	

### Atomic displacement parameters (Å^2^)

**Table:**

	*U*^11^	*U*^22^	*U*^33^	*U*^12^	*U*^13^	*U*^23^
C1	0.0474 (9)	0.0385 (8)	0.0304 (8)	−0.0024 (6)	−0.0002 (7)	0.0035 (6)
C2	0.0595 (11)	0.0423 (9)	0.0505 (11)	−0.0062 (8)	0.0019 (9)	−0.0084 (7)
C3	0.0566 (11)	0.0439 (9)	0.0651 (12)	−0.0167 (8)	0.0002 (9)	−0.0133 (8)
C4	0.0447 (9)	0.0413 (8)	0.0546 (10)	−0.0110 (7)	0.0028 (8)	−0.0001 (7)
C5	0.0438 (9)	0.0341 (7)	0.0354 (8)	−0.0056 (6)	−0.0009 (7)	0.0058 (6)
C6	0.0425 (9)	0.0349 (7)	0.0313 (8)	−0.0050 (6)	−0.0012 (7)	0.0029 (6)
C7	0.0424 (9)	0.0419 (8)	0.0444 (10)	−0.0089 (7)	0.0049 (8)	0.0008 (7)
O8	0.0637 (8)	0.0614 (8)	0.0985 (11)	−0.0251 (6)	0.0386 (8)	−0.0237 (7)
N9	0.0458 (8)	0.0498 (7)	0.0340 (7)	0.0008 (6)	0.0010 (6)	−0.0046 (6)
C10	0.0501 (10)	0.0603 (10)	0.0375 (10)	−0.0003 (7)	−0.0009 (8)	0.0037 (8)
C11	0.0489 (11)	0.0720 (11)	0.0539 (11)	−0.0041 (8)	0.0018 (9)	0.0006 (9)
C12	0.0520 (11)	0.0673 (11)	0.0640 (13)	0.0094 (8)	0.0101 (9)	0.0045 (9)
C13	0.0668 (12)	0.0613 (10)	0.0478 (11)	0.0120 (9)	0.0162 (9)	−0.0037 (9)
C14	0.0614 (11)	0.0528 (9)	0.0457 (11)	0.0040 (8)	0.0032 (9)	−0.0116 (8)

### Geometric parameters (Å, º)

**Table:**

C1—N9	1.3923 (19)	N9—C10	1.4637 (19)
C1—C2	1.398 (2)	C10—C11	1.508 (2)
C1—C6	1.425 (2)	C10—H10A	0.9700
C2—C3	1.368 (2)	C10—H10B	0.9700
C2—H2	0.9300	C11—C12	1.509 (2)
C3—C4	1.373 (2)	C11—H11A	0.9700
C3—H3	0.9300	C11—H11B	0.9700
C4—C5	1.386 (2)	C12—C13	1.514 (2)
C4—H4	0.9300	C12—H12A	0.9700
C5—C6	1.4078 (19)	C12—H12B	0.9700
C5—C7^i^	1.493 (2)	C13—C14	1.509 (2)
C6—C7	1.479 (2)	C13—H13A	0.9700
C7—O8	1.2209 (18)	C13—H13B	0.9700
C7—C5^i^	1.493 (2)	C14—H14A	0.9700
N9—C14	1.4595 (19)	C14—H14B	0.9700
			
N9—C1—C2	120.15 (13)	N9—C10—H10B	109.4
N9—C1—C6	122.30 (13)	C11—C10—H10B	109.4
C2—C1—C6	117.53 (14)	H10A—C10—H10B	108.0
C3—C2—C1	121.86 (15)	C10—C11—C12	110.53 (15)
C3—C2—H2	119.1	C10—C11—H11A	109.5
C1—C2—H2	119.1	C12—C11—H11A	109.5
C2—C3—C4	120.87 (14)	C10—C11—H11B	109.5
C2—C3—H3	119.6	C12—C11—H11B	109.5
C4—C3—H3	119.6	H11A—C11—H11B	108.1
C3—C4—C5	119.62 (15)	C11—C12—C13	109.70 (14)
C3—C4—H4	120.2	C11—C12—H12A	109.7
C5—C4—H4	120.2	C13—C12—H12A	109.7
C4—C5—C6	120.55 (14)	C11—C12—H12B	109.7
C4—C5—C7^i^	116.03 (14)	C13—C12—H12B	109.7
C6—C5—C7^i^	123.40 (12)	H12A—C12—H12B	108.2
C5—C6—C1	119.22 (12)	C14—C13—C12	111.83 (15)
C5—C6—C7	116.76 (13)	C14—C13—H13A	109.3
C1—C6—C7	123.47 (13)	C12—C13—H13A	109.3
O8—C7—C6	122.64 (14)	C14—C13—H13B	109.3
O8—C7—C5^i^	118.49 (13)	C12—C13—H13B	109.3
C6—C7—C5^i^	118.83 (13)	H13A—C13—H13B	107.9
C1—N9—C14	118.71 (12)	N9—C14—C13	110.33 (13)
C1—N9—C10	118.19 (12)	N9—C14—H14A	109.6
C14—N9—C10	111.35 (12)	C13—C14—H14A	109.6
N9—C10—C11	111.04 (13)	N9—C14—H14B	109.6
N9—C10—H10A	109.4	C13—C14—H14B	109.6
C11—C10—H10A	109.4	H14A—C14—H14B	108.1
			
N9—C1—C2—C3	178.97 (15)	C1—C6—C7—O8	5.0 (2)
C6—C1—C2—C3	−2.4 (2)	C5—C6—C7—C5^i^	11.1 (2)
C1—C2—C3—C4	−2.6 (3)	C1—C6—C7—C5^i^	−177.48 (13)
C2—C3—C4—C5	3.7 (3)	C2—C1—N9—C14	14.6 (2)
C3—C4—C5—C6	0.2 (2)	C6—C1—N9—C14	−163.92 (13)
C3—C4—C5—C7^i^	178.57 (15)	C2—C1—N9—C10	−125.26 (15)
C4—C5—C6—C1	−5.3 (2)	C6—C1—N9—C10	56.21 (18)
C7^i^—C5—C6—C1	176.55 (13)	C1—N9—C10—C11	−157.87 (14)
C4—C5—C6—C7	166.56 (14)	C14—N9—C10—C11	59.50 (17)
C7^i^—C5—C6—C7	−11.6 (2)	N9—C10—C11—C12	−57.34 (18)
N9—C1—C6—C5	−175.20 (13)	C10—C11—C12—C13	54.17 (19)
C2—C1—C6—C5	6.2 (2)	C11—C12—C13—C14	−54.10 (19)
N9—C1—C6—C7	13.6 (2)	C1—N9—C14—C13	159.33 (13)
C2—C1—C6—C7	−164.99 (14)	C10—N9—C14—C13	−58.25 (17)
C5—C6—C7—O8	−166.46 (16)	C12—C13—C14—N9	56.03 (18)

Symmetry code: (i) −*x*, −*y*+1, −*z*+1.

### Hydrogen-bond geometry (Å, º)

*Cg*2 is the centroid of the C1–C6 ring.

**Table:**

*D*—H···*A*	*D*—H	H···*A*	*D*···*A*	*D*—H···*A*
C2—H2···*Cg*2^ii^	0.93	2.98	3.850 (2)	156

Symmetry code: (ii) −*x*, *y*−1/2, −*z*+1/2.

### The π–π interaction geometry (Å,°).

**Table:**

*I*	*J*	*CgI*···*CgJ*	Dihedral angle	*CgI*_Perp	*CgJ*_Perp	*CgI*_Offset	*CgJ*_Offset
2	2^ii^	3.806 (1)	0	3.702 (1)	3.702 (1)	0.884 (1)	0.884 (1)

Symmetry code: (ii) –*x*, –*y*, –*z* + 1.Notes: *Cg*2 is the centroid of the C1–C6 ring. Cg*I*···Cg*J* is the distance between ring centroids. The dihedral angle is that between the planes of the rings *I* and *J*. *CgI*_Perp is the perpendicular distance of *CgI* from ring *J*. *CgJ*_Perp is the perpendicular distance of *CgJ* from ring *I*. Cg*I*_Offset is the distance between *CgI* and perpendicular projection of *CgJ* on ring *I*. Cg*J*_Offset is the distance between *CgJ* and perpendicular projection of *CgI* on ring *J*.
